# *De Novo* Generation of a Unique Cervid Prion Strain Using Protein Misfolding Cyclic Amplification

**DOI:** 10.1128/mSphere.00372-16

**Published:** 2017-01-25

**Authors:** Crystal Meyerett-Reid, A. Christy Wyckoff, Terry Spraker, Bruce Pulford, Heather Bender, Mark D. Zabel

**Affiliations:** Prion Research Center at Colorado State University, Department of Microbiology, Immunology and Pathology, College of Veterinary Medicine and Biomedical Sciences, Fort Collins, Colorado, USA; University of Michigan

**Keywords:** chronic wasting disease, prions, protein misfolding cyclic amplification, spontaneous generation, strains

## Abstract

Substantial evidence supports the hypothesis that prions are misfolded, infectious, insoluble, and protease-resistant proteins (PrP^RES^) devoid of instructional nucleic acid that cause transmissible spongiform encephalopathies (TSEs). Protein misfolding cyclic amplification (PMCA) has provided additional evidence that PrPRes acts as a template that can convert the normal cellular prion protein (PrP^C^) present in uninfected normal brain homogenate (NBH) into the infectious misfolded PrP^RES^ isoform. Human PrP^C^ has been shown to spontaneously convert to a misfolded pathological state causing sporadic Creutzfeldt-Jakob disease (sCJD). Several investigators have reported spontaneous generation of prions by *in vitro* assays, including PMCA. Here we tested the rate of *de novo* generation of cervid prions in our laboratory using our standard PMCA protocol and NBH from transgenic mice expressing cervid PrP^C^ (TgCerPrP mice). We generated *de novo* prions in rounds 4, 5, and 7 at low cumulative rates of 1.6, 5.0, and 6.7%, respectively. The prions caused infectious chronic wasting disease (CWD) upon inoculation into normal uninfected TgCerPrP mice and displayed unique biochemical characteristics compared to other cervid prion strains. We conclude that PMCA of cervid PrP^C^ from normal brain homogenate spontaneously generated a new cervid prion strain. These data support the potential for cervids to develop sporadic CWD.

**IMPORTANCE** CWD is the only known TSE that affects free-ranging wildlife, specifically cervids such as elk, deer, moose, caribou, and reindeer. CWD has become endemic in both free-ranging and captive herds in North America, South Korea, and, most recently, northern Europe. The prion research community continues to debate the origins of CWD. Original foci of CWD emergence in Colorado and Wyoming coincident with the sheep TSE scrapie suggest that scrapie prions may have adapted to cervids to cause CWD. However, emerging evidence supports the idea that cervid PrP^C^ may be more prone to misfolding to the pathological isoform. Here we test the hypothesis that cervid PrP^C^ can spontaneously misfold to create *de novo* prions. Whether CWD can arise spontaneously as a sporadic TSE or represents a new TSE caused by cervid-adapted scrapie prions profoundly impacts surveillance and mitigation strategies.

**Podcast**: A podcast concerning this article is available.

## Abstract (precis)

CWD is the only known TSE that affects free-ranging wildlife, specifically cervids such as elk, deer, moose, caribou, and reindeer. CWD has become endemic in both free-ranging and captive herds in North America, South Korea, and, most recently, northern Europe. The prion research community continues to debate the origins of CWD. Original foci of CWD emergence in Colorado and Wyoming coincident with the sheep TSE scrapie suggest that scrapie prions may have adapted to cervids to cause CWD. However, emerging evidence supports the idea that cervid PrP^C^ may be more prone to misfolding to the pathological isoform. Here we test the hypothesis that cervid PrP^C^ can spontaneously misfold to create *de novo* prions. Whether CWD can arise spontaneously as a sporadic TSE or represents a new TSE caused by cervid-adapted scrapie prions profoundly impacts surveillance and mitigation strategies.

## INTRODUCTION

Prions cause diseases classified as transmissible spongiform encephalopathies (TSEs), which are characterized by distinct neuropathology, including neuronal loss, vacuolation, and astrogliosis, that eventually causes behavioral and cognitive defects and death (1–3). Bovine spongiform encephalopathy (BSE) of cattle, Creutzfeldt-Jakob disease (CJD) of humans, scrapie of sheep and goats, and chronic wasting disease (CWD) of cervids (deer, elk, moose, caribou, and reindeer) are notable prion diseases that can be transmitted or inherited or can occur spontaneously. Mounting evidence supports the prion hypothesis that TSE pathogenesis is caused by the conversion of the normal host cellular prion protein (PrP^C^) into a protease-resistant, abnormal disease-causing isoform devoid of instructional nucleic acid (PrP^RES^) (4–7).

*In vitro* generation of infectious prion protein using protein misfolding cyclic amplification (PMCA) has substantiated the protein-only hypothesis. PMCA of prions occurs when a prion seed templates misfolding of PrP^C^ present in uninfected normal brain homogenate (NBH) into the pathological, infectious isoform (8). Short bursts of sonication break up prion aggregates, creating more templates for further conversion of PrP^C^ to PrP^RES^ (9). Employing repeated cycles of incubation and sonication has led to efficient amplification of minute quantities of PrP^RES^ using substrates from various species (9–12). Employing serial PMCA (sPMCA), or rediluting PMCA products into fresh NBH after a given number of rounds and continuing PMCA, has resulted in successful evaluations of strain adaptation and species barriers in the absence of lengthy and expensive bioassays (9, 13–16). Deleault et al. identified the minimal PMCA requirements for *in vitro* amplification resulting in spontaneous generation of infectious prions from noninfectious components: native hamster PrP^C^ in combination with copurified lipids and synthetic polyanions (17). Barria et al. expanded on these findings and reported generating spontaneous mouse and hamster prions from uninfected normal brain homogenate substrate in a prion-free laboratory upon extended rounds and modification of normal sPMCA conditions (18).

The origins of CWD are openly debated. Discovery of the first CWD foci in areas of Colorado and Wyoming with relatively sparse populations of cervids suggests to some transmission of sheep scrapie, present in those locations, to deer and elk grazing nearby and perhaps gaining access to contaminated sheep pens (19). However, the possibility of spontaneously generated cervid prions causing sporadic CWD has not been excluded. Recently, the first natural cases of CWD in Europe were detected in Norwegian reindeer and moose that had no evident contact with animals or areas exposed to prions (20). Mounting evidence suggests that amino acids 170 to 174 of cervid PrP^C^ form a rigid loop which increases the propensity for spontaneous misfolding (21–24). In order to detect and examine the rate of spontaneous conversion of cervid PrP^C^, we subjected unseeded, uninfected brain homogenate derived from cervid PrP^C^-expressing transgenic mice (TgCerPrP^C^ mice) to seven rounds of PMCA with 48 cycles per round. In order to avoid the possibility of cross-contamination, all experiments were performed in a prion-free laboratory with new reagents and equipment. Here we report for the first time detection of protease K (PK)-resistant cervid PrP^C^ by Western blotting (WB) at round 4 of sPMCA at a rate of 1.6%. Further generation of spontaneous prions was observed after round 5 and round 7, with rates of 5.0% and 6.74%, respectively. Bioassay determined that the spontaneously generated cervid prions were infectious to cervidized transgenic mice but not wild-type (WT) mice, and biochemical analysis revealed a unique prion profile differing from the profiles seen with prion strains used within our laboratory. These data strongly suggest that those prions arose spontaneously and not from contamination. We propose that the *de novo* generation of infectious cervid prions in our laboratory by PMCA spontaneously created a novel cervid prion strain, supporting the idea that cervid PrP^C^ can spontaneously convert to cervid prions *in vivo* and the possibility of sporadic CWD.

## RESULTS

### Generation of *de novo* cervid prions using PMCA.

We have previously reported that NBH from transgenic mice expressing cervid PrP^C^ (TgCerPrP mice) supports efficient PMCA amplification using a standard PMCA protocol (15, 25, 26). While false positives in our negative controls have been rare, even under conditions of strict measures the possibility of contamination cannot be dismissed. The rare false positives in our negative controls are primarily observed after 5 rounds of PMCA and occur sporadically, while true positives are generally observed by 3 rounds in multiple replicates. The late rounds at which we observed false positives led us to hypothesize that they arose from spontaneous misfolding of cervid PrP^C^ rather than contamination. To test this hypothesis and determine the rate of PMCA false positives in our laboratory, we ran unseeded TgCerPrP mouse NBH through our standard sPMCA protocol. Three groups of 20 NBH samples were subjected to 8 rounds of sPMCA in a prion-free laboratory to avoid cross-contamination. Additionally, all reagents and equipment were new and brain homogenate was made in a different prion-free laboratory. After each round of PMCA (48 cycles of 30 min of incubation followed by 40 s of sonication), NBH samples were analyzed by protease K (PK) digestion and Western blotting, and any confirmed positives were removed from the PMCA plate to avoid contamination of the remaining negative samples.

**FIG 1:**
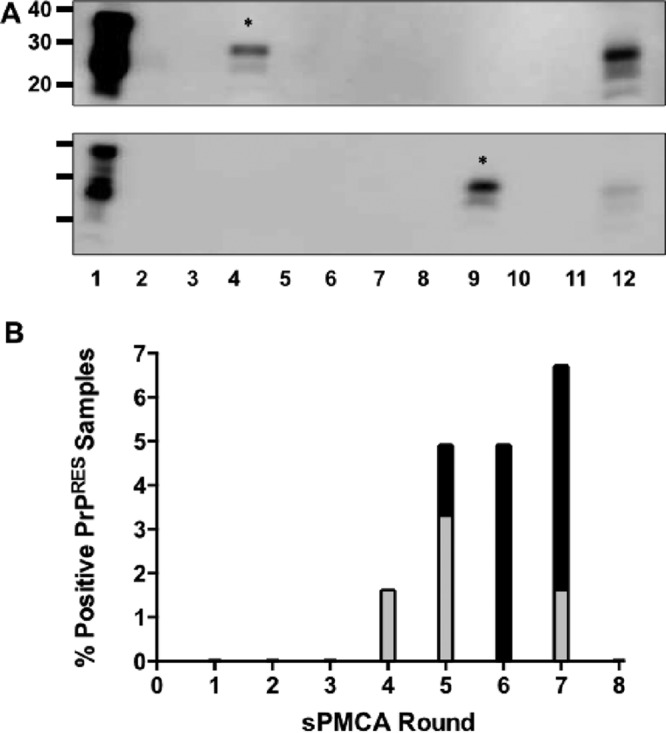
*De novo* generation of cervid prions. (A) Representative Western blots of *de novo* prions generated by sPMCA. Samples in all lanes were digested with 50 µg/ml proteinase K except lane 1, and all lanes contain NBH amplified by PMCA except lane 12, which contains unamplified deer prions for comparison. *De novo* prions are indicated by an asterisk (*). (B) Rate of *de novo*-generated prions at the respective rounds. Gray bars indicate the rate of *de novo* generation for that individual round, and black bars indicate the cumulative rate.

Western blot analysis resulted in identification of one protease-resistant PrP (PrP^RES^) sample after 4 rounds of sPMCA (Fig. 1A) which generated *de novo* PrP^RES^ at a rate of 1.7% that increased to cumulative rates of 5.0% and 6.7% after five and seven rounds of sPMCA, respectively (Fig. 1B).

### *De novo* cervid prions are infectious.

**FIG 2:**
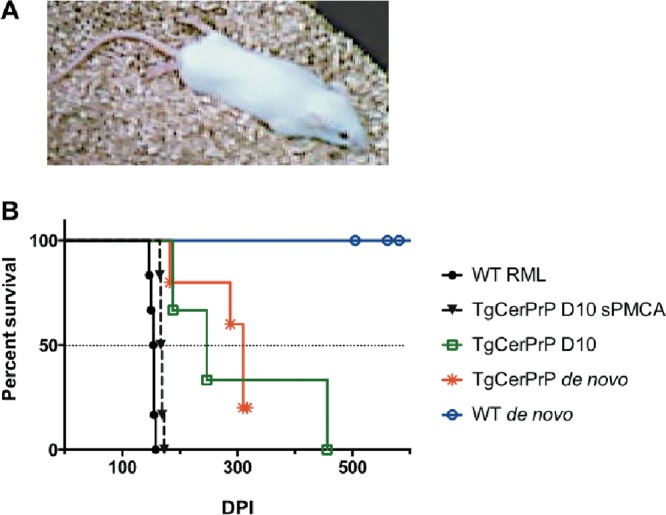
*De novo* prions are infectious. (A) Photo of one mouse representative of TgCerPrP mice inoculated with *de novo* prions afflicted with terminal CWD. Note the hind limbs extended behind the mouse, indicating paralysis. (B) TgCerPrP mice inoculated with *de novo* prions died from terminal disease at a median of 300 dpi, while WT mice remained disease free for the rest of their lives (>500 dpi). TgCerPrP mice inoculated with D10 died approximately 50 days earlier, while TgCerPrP mice inoculated with sPMCA-adapted prions and WT mice inoculated with RML prions died over 100 days earlier.

To determine if *de novo*-generated prions were infectious, we intracerebrally (i.c.) inoculated TgCerPrP and WT mice with PK-resistant and Western blot-positive *de novo* prions generated by PMCA. Here we report that TgCerPrP mice but not WT mice inoculated with *de novo* prions displayed clinical signs of disease. Four of five TgCerPrP mice inoculated with *de novo* prions died by 317 days postinoculation (dpi), with a mean time to terminal disease of 273 ± 61 dpi (Table 1 and Fig. 2). We observed subclinical disease and identical strain characteristics in the fifth TgCerPrP mouse by biochemical and neuropathological analysis and include that mouse in subsequent prion strain characterization. *De novo* prion-infected TgCerPrP mice lost 15% of body weight and exhibited stiff tails, ataxia, and impaired hind limb extensor reflex (Fig. 2A). None of five WT mice inoculated with *de novo* cervid prions showed any signs of prion disease or infectivity in their brains after 500 dpi (Table 1). The disease course of *de novo*-infected TgCerPrP mice was comparable to that of TgCerPrP mice inoculated with the D10 cervid prion isolate (285 ± 55 dpi) but occurred over 100 days later than that seen with TgCerPrP mice inoculated with D10 that was adapted to mice via sPMCA using TgCerPrP brain homegenate (169 ± 4 dpi) or WT mice inoculated with mouse-adapted Rocky Mountain Laboratory (RML) scrapie (155 ± 6 dpi, Fig. 2B). The variance in the results with respect to the number of days from infection to terminal disease, measured by the standard deviation of the mean, was much larger in mice inoculated with the D10 primary isolate (55 dpi) or *de novo* prions (61 dpi) than in mice inoculated with adapted prion strains (4 to 6 dpi).

**TABLE 1:** Summary of inoculation experiments comparing *de novo* incidence and incubation period to previously reported prion strains from our laboratory (15)

Inoculum^a^	Mouse strain			
	Cervidized transgenic		Wild type	
	Incidence^b^	DPI + SD^c^	Incidence	DPI + SD
*De novo*	4/5	273 ± 61	0/5	>500^d^
D10^f^	8/8	285 ± 55	0/5	>500
RML^f^	ND^e^	ND	6/6	155 ± 6
sPMCAD10^f^	6/6	169 ± 4^d^	ND	ND

a Mice were inoculated intracerebrally.

b Incidence = number of terminally sick mice/number of animals inoculated.

c DPI ± SD, days postinoculation to terminal disease ± standard deviation.

d *P* < 0.01 compared to *de* *novo*.

e ND, no data.

f Previously reported data.

### *De novo* cervid prions display and propagate distinct glycoform profiles.

**FIG 3:**
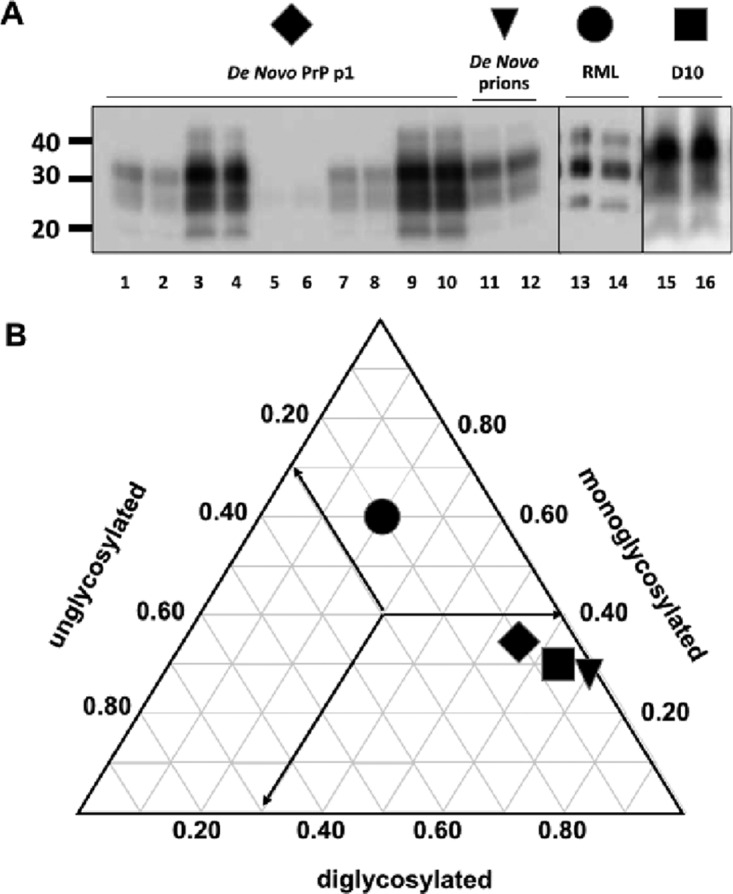
*De novo* prions display and propagate distinct glycoform profiles. (A) Representative Western blots of protease K-digested brain homogenates from mice infected with *de novo* prions (lanes 1 to 10), the *de novo* prions used as the inoculum (lanes 11 and 12), RML (lanes 13 and 14), and D10 (lanes 15 and 16). Duplicate samples were analyzed after PK digestion, and glycoform ratios were determined by densiometric analysis. (B) Triplot of mean glycoform ratios. Arrows indicate the direction of the axis for each glycosylation type. *De novo* prions (triangle) and D10 prions (square) were predominantly diglycosylated, while prions from TgCerPrP mice inoculated with *de novo* prions (diamond) exhibited increased levels of underglycosylation. All cervid prions appeared distinct from the predominantly monoglycosylated pattern exhibited by RML prions (circle). Bars indicating standard deviation from the mean do not extend beyond the symbols.

We also assessed the biochemical differences of *de novo*-generated prions from other laboratory prion strains. Glycoform ratios have been a useful tool for characterizing different prion strains as evidenced by the presence of a predominant diglycosylated band in CWD prions compared to a predominant monoglycosylated band of scrapie prions (15). We compared glycoform ratios of PK-digested *de novo* prions, *de novo* prions passaged through TgCerPrP mice, and D10 and RML prions by densitometric analysis of Western blots (Fig. 3A). *De novo* prions contained predominantly diglycosylated PrP^RES^ (70 ± 3%), less monoglycosylated PrP^RES^ (29 ± 2%), and virtually no unglycosylated PrP^RES^ (1 ± 1%, *n* = 5, *P* < 0.01), clearly distinct from the D10 glycoform profile (63 ± 3% di-, 30 ± 2% mono-, and 7 ± 1% unglycosylated, *P* < 0.05, Fig. 3B). Prions generated upon passage of *de novo* prions into TgCerPrP mice (*de novo* p1) displayed a glycoform profile trending toward a more balanced ratio of diglycoforms (57 ± 2%) and monoglycoforms (37 ± 3%) reminiscent of but still distinct from the ratios seen with RML (20 ± 1% di-, 60 ± 2% mono-, and 20 ± 1% unglycosylated), D10, and the original *de novo* inoculum (*P* < 0.01).

### *De novo* cervid prions display and propagate distinct conformational stabilities.

**FIG 4:**
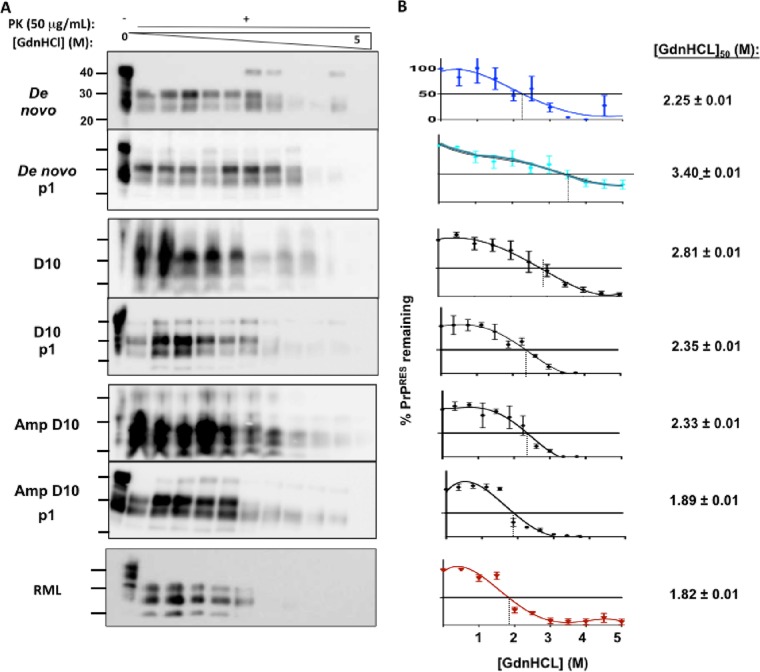
*De novo* prions display and propagate distinct conformational stabilities. We assessed three replicates of brain homogenates from all mice in each group (*n* = 5). (A) Western blots of brain extracts denatured with increasing guanidine hydrochloride (GdnHCl) concentrations and treated with 50 μg/ml·PK. (B) Corresponding denaturation curves quantify remaining PrP^RES^. To the right of each curve is the concentration of GdnHCl required to denature 50% of the PrP^RES^ [(GdnHCl)_50_]. *De novo* prions exhibited conformational stability similar to that of D10 prions serially amplified (Amp D10) or passaged into cervidized mice (D10 p1), but the corresponding data were statistically significantly different from D10 data (*P* < 0.01). Passage of *de novo* prions into cervidized mice produced prions (*de novo* p1) whose incredibly stable conformation was much greater than that seen with the original *de novo* prions, while passaging the original D10 (D10 p1) or amplified D10 (Amp D10 p1) through mice produced prions with comparatively decreased stability.

We further assessed *de novo* prion strain characteristics biochemically by denaturing prion-positive brain homogenate with increasing concentrations of guanidine hydrochloride (GdnHCl), followed by PK digestion and Western blot analysis of the remaining PrP^RES^ (Fig. 4A). *De novo* prions exhibited a conformational stability [mean (GdnHCl)_50_ value, 2.25 ± 0.01 M] that was similar to that of D10 prions passaged through cervidized mice (D10p1) (2.35 ± 0.01 M) or serially amplified by PMCA (Amp D10) (2.33 ± 0.01 M) but was significantly different from D10 results (Fig. 4B, P < 0.001). Passage of *de novo* prions through TgCerPrP mice (*de novo* p1) resulted in a significantly higher (GdnHCl)_50_ value of 3.40 ± 0.01 M compared to all other strains, including amplified D10 prions passaged into cervidized mice (Amp D10 p1, 1.89 ± 0.01 M, *P* < 0.001).

### *De novo* cervid prions cause distinct neuropathology and generate unique lesion profiles.

**FIG 5:**
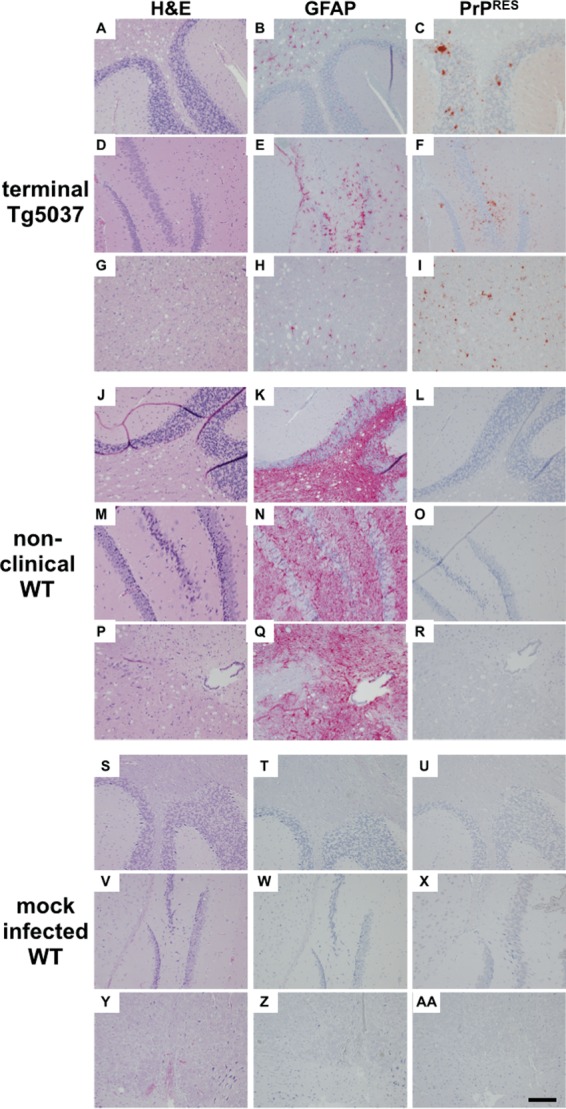
*De novo* prions cause distinct neuropathology. Brain sections from all mice in each group (*n* = 5), including terminally sick TgCerPrP mice inoculated with *de novo* prions (panels A to I), nonclinical WT mice (J to R), and mock-infected WT mice (S to AA), were assessed for vacuolation (column 1; H&E), astrogliosis (column 2; GFAP), and PrP^RES^ deposition (column 3; PrP^RES^) in the cerebellum (A to C, J to L, and S to U), the hippocampus (D to F, M to O, and V to X), and the medulla (G to I, P to R, and Y to AA). TgCerPrP mice exhibited dense punctate PrP^RES^ plaques in all three areas, whereas PrP^RES^ staining was absent from WT and control mice. Dense GFAP staining was identified in aged, nonclinical WT mice but was less prominent and more focal in terminal TgCerPrP mice. Vacuolation was observed in WT and TgCerPrP mice but was absent or less intense in the hippocampus. Data from mock-infected WT mice at >300 dpi serve as negative controls. Images shown are representative of all mice in each group. Scale bar, 50 µm.

Next we assessed vacuolation, astrogliosis, and PrP^RES^ deposition in brain areas of WT and TgCerPrP mice inoculated with *de novo* prions (Fig. 5). Brains of WT mice inoculated with *de novo* prions exhibited larger amounts of astrogliosis and vacuolation (Fig. 5J to R) than those of *de novo* prion-inoculated TgCerPrP mice (Fig. 5A to I) and negative-control mice (Fig. 5S to AA). Interestingly, astrogliosis in WT mice was diffuse and prominent throughout all brain sections (Fig. 5K and N and Q). In contrast, TgCerPrP mice exhibited much lower levels of reactive astrocytes in analyzed sections (Fig. 5B and E and H) but higher levels than negative controls (Fig. 5T and W and Z). Small amounts of dense punctate prion deposits, characteristic of cervid prions, were identified in brain sections of *de novo* prion-inoculated TgCerPrP mice (Fig. 5C and F and I) but were absent in inoculated WT mice (Fig. 5U and X and AA). Prominent PrP^RES^ accumulation was consistently observed in the cerebellum (Fig. 5C), the hippocampus (Fig. 5F), and the dorsal medulla (Fig. 5I) in *de novo* prion-inoculated TgCerPrP mice. PrP^RES^ deposits were located within the granular cells within the cerebellum of TgCerPrP mice, while vacuolation was prominent in the white matter of the arbor vitae. WT mice inoculated with *de novo* prions did not develop CWD or exhibit PrP^RES^ staining but exhibited appreciable vacuolation and abundant reactive astrocytes.

**FIG 6:**
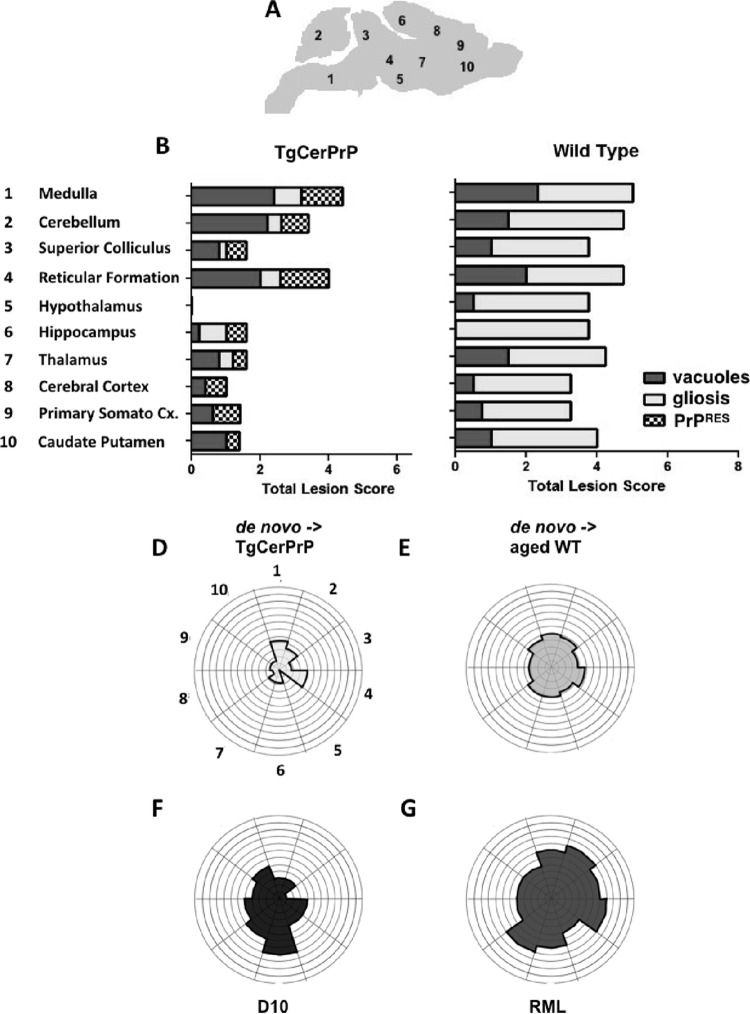
*De novo* prions generate unique lesion profiles. (A) Levels of neuropathology of 10 brain areas from all mice in each group (*n* = 5) were given scores of 0 to 4 (0 indicating no neuropathology and 4 indicating severe neuropathology) for vacuolation (H&E), astrogliosis (GFAP), and PrP^RES^ deposition. (B and C) Scores were summed and graphed for *de novo* prion-inoculated, terminally sick TgCerPrP mice (B) and nonclinical WT mice (C). Separation of lesion scores by criteria revealed significant PrP^RES^ deposition in TgCerPrP mice that was absent in WT mice. Conversely, GFAP staining indicative of astrogliosis was relatively mild in TgCerPrP mice compared to the massive GFAP staining observed in nonclinical aged WT mice. Vacuolation scores of TgCerPrP and WT mice were similar. Cx., cortex. (D to G) Summed scores graphed on radar plots produced composite lesion profiles for comparisons. TgCerPrP mice inoculated with *de novo* prions (D) exhibited a unique profile with relatively less severe neuropathology overall than nonclinical aged WT mice inoculated with *de novo* cervid prions (E), terminally sick TgCerPrP mice inoculated with D10 (F), or RML-inoculated WT mice (G) (15).

We assessed neuropathology in seven additional brain areas (Fig. 6A) and generated a composite lesion profile score by summing scores for vacuolation, astrogliosis, and PrP^RES^ deposition for all areas in *de novo* prion-inoculated mice (Fig. 6B and C). TgCerPrP mice had little to no GFAP staining in brain areas (Fig. 6B), but vacuoles were present in patterns similar to those seen with WT mice (Fig. 6C). PrP^RES^ deposition was predominant in the reticular formation, medulla, and cerebellum but was completely absent in the hypothalamus of *de novo* prion-inoculated TgCerPrP mice (Fig. 6B and D). WT mice exhibited intense GFAP staining in all brain areas and moderate vacuolation in these areas except the hippocampus (Fig. 6C and E). However, WT mice accumulated no detectable PrP^RES^ anywhere in their brains.

Analysis of total lesion profile scores represented in radar plots (Fig. 6 and D to G) revealed *de novo*-inoculated TgCerPrP mice that displayed clinical signs of disease and had PrP^RES^ present in most brain areas and yet exhibited less overall neuropathology (Fig. 6B and D) than mice in other cohorts (Fig. 6C and E to G). WT mice inoculated with *de novo* prions had a composite lesion profile (Fig. 6E) similar to that seen with RML-inoculated WT mice (Fig. 6G), even though *de novo*-inoculated WT mice were not clinically sick and accumulated no PrP^RES^ plaques even after >500 dpi (Fig. 6C).

## DISCUSSION

Evidence supporting the idea of spontaneous generation of prions causing sporadic Creutzfeldt-Jakob disease (sCJD) in humans (27–29) also supports related hypotheses about the inception of some animal prion diseases such as chronic wasting disease and scrapie. But distinguishing between a spontaneous prion event and infectious transmission in wild populations, such as cervids afflicted with CWD, for example, remains quite difficult. Carefully controlled laboratory experiments using transgenic mouse models and *in vitro* assays help tease apart these prionogenic pathways and provide valuable information concerning spontaneous prion generation.

Several prion research groups have reported the *in vitro* generation of *de novo* prions using PMCA (17, 18) and synthetic prions using recombinant PrP (30–32) and, inadvertently, by binding PrP^C^ and presumable cofactors to metal (30). Previous publications of *de novo* generation of hamster or mouse PrP^RES^ utilizing PMCA reported the requirement of synthetic polyanions and/or copurified lipids and reported modification of PMCA by increasing incubation and sonication cycles (17, 18). Here we report generation of *de novo* prions at a low frequency by standard PMCA methods, using normal brain homogenate in a prion-free environment.

Barria et al. reported the occurrence of spontaneous conversion of human, mouse, and hamster PrP^C^ substrates using PMCA only by extending the number of PMCA cycles and rounds, where 240 cycles (5 days) constituted one round (18). These substrates did not generate *de novo* prions by standard PMCA protocols. Similar results were obtained in experiments using hamster substrate with addition of polyanions (33). Here we generated *de novo* prions using cervid PrP^C^ substrate relatively easily using our standard PMCA protocol without additional PMCA cofactors, cycles, or rounds, suggesting that cervid PrP^C^ misfolds more easily than human, hamster, or mouse PrP^C^. Altering the flexible loop that connects the β-2 strand and α2 helix of mouse PrP to the rigid loop found in cervid PrP^C^ via amino acid substitutions S170N and N174T resulted in spontaneous prion disease *in vivo* (22), while changing the rigid loop structure from a 3_10_- to a type I β-helix turn by introducing a Y169G mutation prevented prion disease (34). Additionally, cell-free misfolding and conversion assays of recombinant mouse PrP containing cervid amino acid substitutions that form the rigid loop had a higher propensity of misfolding than assays using WT recombinant PrP (24). We hypothesize that the unique rigid loop in the globular domain of cervid PrP^C^ facilitates generation of spontaneous cervid prions by promoting thermodynamic instability and pathogenic misfolding. A recent report that CWD prions passaged either *in vitro* by PMCA or *in vivo* through TgCerPrP mice were able to template misfolding of human PrP^C^ by PMCA (35) supports this hypothesis and implies that the rigid loop of cervid PrP^C^ may contribute not only to spontaneous prion generation but also to facile strain adaptation. *In toto*, these data highlight a propensity of cervid PrP^C^ to spontaneously misfold that could explain the seemingly facile transmission of CWD among cervids and portend significantly weakened species barriers as CWD prions evolve.

We characterized the *de novo* prions biologically, biochemically, and neuropathologically and compared them to other prion strains. We observed an incomplete attack rate and a delay in median time to terminal disease in TgCerPrP mice inoculated with *de novo* prions compared to D10 and a D10-seeded sPMCA product (sPMCA D10). WT mice were not susceptible to any of those cervid strains and contained no PrP^RES^ in their brains by either PK analysis and WB or immunohistochemistry (IHC), indicating that the *de novo* prions were indeed cervid prions. We have previously shown that PMCA adapts prions *in vitro* as effectively as in *in vivo* passage in TgCerPrP mice (15). If *de novo* prions were identical to D10 prions or arose from contamination, we would expect transmission to susceptible mice to be as efficient as that seen with mouse-passaged D10 and sPMCA D10.

Biochemical and neuropathological lesion profiling also supports the idea of spontaneous generation of a new cervid prion strain. The glycoform profile and conformational stability of *de novo* prions were markedly different before and after passage through TgCerPrP mice from those of the other prion strains that we analyzed. The glycoform profile of *de novo* prions drifted toward a more balanced ratio of mono- and diglycosylated PrP^Sc^. We also noticed a slight increase in conformational stability in the presence of 2 to 3 M GdnHCl, with the data appearing as a slight “hump” in the middle of the graph (Fig. 4). While quite subtle, it could indicate biphasic denaturation unique to *de novo* prions. We occasionally see a PK-resistant, electrophoretically unshifted product, indicative of a full-length, PK-resistant PrP species, in our negative-control PMCA samples. We suspect that this may be an intermediate form of PrP as it transitions from PrP^C^ to PrP^Sc^, causing the anomalous denaturation curve. We are currently investigating this possibility, as similar intermediate PrP species have been proposed previously (30). We are also assessing whether the strain adaptation trend continues and/or convergence of other strain characteristics toward an RML phenotype occurs upon further passage through mice. We exclude RML contamination contributing to these results because we used new facilities and equipment that had never been exposed to prions to generate *de novo* prions. We did not introduce RML into any of these rooms or equipment. The vastly different glycoform ratios and conformational stabilities of RML versus *de novo* prions also argue against RML or any other prion contamination accounting for our results.

In marked contrast to the results seen with primary CWD prion isolates or adapted CWD prion strains, inoculation of *de novo* prions into TgCerPrP mice generated a prion strain with greatly increased conformational stability, which may contribute to the observed increased incubation time and decreased mortality (36, 37). Neuropathologic analysis of TgCerPrP mice inoculated with *de novo* prions showed dense punctate plaques of PrP^RES^ characteristic of CWD that were absent in WT and mock-infected controls. Curiously, WT mice inoculated with *de novo* cervid prions exhibited intense astrogliosis and significant vacuolation in the absence of PrP^RES^ deposition and clinical signs of terminal disease. These criteria skewed the WT lesion profile score such that it exceeded that of terminally sick *de novo*-inoculated TgCerPrP mice. The age of control mice at time of necropsy likely contributed to the observed neuropathology. Perhaps WT mice were subclinical carriers of CWD prions at study termination. *De novo* cervid prions may adopt a conformation *in vivo* that masks the epitope of the antibodies we used for IHC, although we failed to detect any PrP^RES^ using the same antibodies in our denaturing Western blots of brain homogenates from the same animals. Castilla et al. reported that a species barrier between Tg (porcine) mice and low-dose BSE prions resulted in a subclinical infection that became evident only after repassage into Tg (porcine) mice (38). We are currently exploring this possibility by serial passage of brain homogenate from nonclinical WT mice into additional WT mice.

In summary, we have generated with relative ease by PMCA a *de novo* CWD prion strain with novel biological, biochemical, and neuropathological features. These data provide further evidence that cervid PrP^C^ exhibits tertiary structural plasticity that we hypothesize contributes to a higher propensity for misfolding and potentially weakened species barriers that could promote interspecies transmission.

## MATERIALS AND METHODS

### Preparation of normal brain homogenate (NBH).

All NBH preparations were completed in a laboratory never previously exposed to prions. Mice were euthanized and perfused with 30-ml 5 mM EDTA–phosphate-buffered saline (PBS). Whole brains were removed and immediately frozen in liquid nitrogen. Brains were weighed and transferred into 1.5-ml Eppendorf tubes containing 2.5-mm-diameter glass beads. PMCA buffer (150 mM NaCl, 50 mM EDTA, PBS) with 2× Complete protease inhibitor cocktail (Roche) was added to make a 20% (wt/vol) solution. Samples were homogenized for 20 s at 4.5 m/s in a FastPrep machine (Biogene), cooled on ice for 2 min, and centrifuged at 14,000 × *g* for 10 s to reduce foaming. This process was repeated twice. NBH was diluted to a 10% (wt/vol) solution by adding an equal volume of PMCA conversion buffer containing 2% Triton X-100 and was incubated on ice for 20 min. NBH was clarified by centrifugation for 5 min at 1,500 × *g*, and supernatants were aliquoted into new tubes and stored at −70°C.

### Serial PMCA.

sPMCA was conducted in a new prion-free laboratory separate from the laboratory in which the NBH was made. All reagents and equipment were new and had never been used in a prion-contaminated laboratory. sPMCA was modified from a previous protocol (15). Briefly, 50-µl samples of 10% NBH were placed into 20 wells of a 96-well microplate. The entire plate was suspended in the water bath of a new 3000MP sonicator (Misonix, Inc.) and sonicated at 70 to 85% maximum power for 40 s, followed by a 30-min incubation at 37°C. This cycle was repeated 48 times, constituting 1 round. For each additional round, 25 µl of NBH from the previous round was diluted into 25 µl of fresh NBH and subjected to another PMCA round. This process was repeated for a total of 8 rounds. Replicate experiments of 20 NBH samples were started 3 days apart, totaling 3 experiments and 60 NBH samples. Gloves were changed after each replicate group experiment, and Western blot analyses of the samples were performed immediately after the completed round to look for PK-resistant material. Any PK-resistant material that was definitively positive was not subjected to further rounds of PMCA to avoid cross-contamination of other samples. Those samples that were not definitively positive (low band intensities and banding patterns that mimicked those of undigested material) were subjected to another round of PMCA to confirm positivity. If the samples also showed positivity at the next round, the results were called positive at the previous round.

### Mice.

B6129SF2/J mice (stock number 101045) were purchased from the Jackson Laboratories (Bar Harbor, ME). Tg(CerPrP)5037 mice were generated as previously described (39). All mice were bred and maintained at Lab Animal Resources, accredited by the Association for Assessment and Accreditation of Lab Animal Care International, in accordance with protocols approved by the Institutional Animal Care and Use Committee at Colorado State University.

### Sources and preparation of prion inocula.

Brain homogenates (10%) were prepared in PMCA buffer (4 mM EDTA, 150 mM NaCl, PBS). PMCA samples that were Western blot positive, indicating the presence of *de novo* prions, were pooled to be used as the inoculum. We diluted inoculum 1:10 in 320 mM sucrose supplemented with 100 units/ml penicillin and 100 µg/ml streptomycin (Gibco) in PBS 30 min prior to intracerebral inoculations. The remaining *de novo* brain homogenate from PMCA was further amplified to 10 rounds in order to create enough *de novo* positive brain material for biochemical analysis.

### PK digestion and Western blotting.

Samples were digested with 50 μg/ml·PK (Roche) for 30 min at 37°C. The reaction was stopped by adding lithium dodecyl sulfate sample loading buffer (Life Technologies, Inc.) and incubating at 95°C for 5 min. Proteins were electrophoretically separated through 12% sodium dodecyl sulfate-polyacrylamide gels (Invitrogen), and transferred to polyvinylidene difluoride membranes (Millipore). Nonspecific membrane binding was blocked by incubation in 5% milk blocking solution (Bio-Rad) for 1 h. Membranes were then incubated for 1 h at room temperature with horseradish peroxidase-conjugated Bar224 anti-PrP monoclonal antibody (SPI bio) diluted 1:20,000 in Superblock (Pierce), washed six times for 10 min each time in PBS–0.2% Tween 20, and incubated for 5 min with enhanced chemiluminescent substrate (Millipore). Membranes were digitally photographed using a FujiDoc gel documentation system equipped with a cooled charge-coupled diode camera (Fuji). Densitometric analyses were performed using Quantity One software (Bio-Rad).

### Prion inoculations and clinical scoring.

Mice were anesthetized by isofluorane inhalation. Thirty microliters of the inoculum was injected intracerebrally 3 mm deep through the coronal suture at a location 3 to 5 mm lateral to the sagittal suture. Mice were monitored daily for clinical symptoms of prion disease, including tail rigidity, impaired extensor reflex, akinesia, tremors, ataxia, 15% weight loss, and paralysis. Mice exhibiting any four of these symptoms or paralysis were scored terminally sick and euthanized.

### Histochemistry and immunohistochemistry (IHC).

Tissues were fixed in 10% paraformaldehyde and embedded in paraffin, and 5- to 10-µm-thick sections were cut and mounted on glass slides. For PrP staining, tissue sections were deparaffinized, treated with concentrated formic acid for 30 min, and then autoclaved at 121°C in target retrieval solution (Dako) for 2 h, washed twice for 7 min each time in 1× PBS, treated with 0.3% H_2_O_2_–methanol for 30 min, and blocked for 1 h with 5% bovine serum albumin (BSA)–PBS and mixed 1:1 in Superblock (Pierce). Excess block solution was tapped off, and sections were incubated with anti-PrP Bar 224 monoclonal antibody, recognizing PrP epitope from amino acids 141 to 151 (human sequence), diluted 1:500 in block solution for 1 h. Slides were then washed in PBS twice for 7 min each time and incubated 30 min with Envision plus horseradish peroxidase (HRP) mouse secondary antibody (Dako). After another two 7-min washes, slides were incubated for 5 to 7 min with AEC+ substrate-chromagen (Dako) and rinsed in PBS twice for 7 min each time and counterstained with hematoxylin. Slides were rinsed in H_2_O, immersed in a 0.1% sodium bicarbonate bluing reagent for 5 min, rinsed in tap water, and mounted on coverslips with aqueous mounting medium (Richard Allan Scientific). Hematoxylin and eosin (H&E) and glial fibrillary acidic protein (GFAP) staining was performed on a NexES automated IHC stainer (Ventana Medical Systems, Inc. Tucson, AZ). Sections were stained with H&E for 4 min at room temperature. Sections were stained with rabbit polyclonal antisera against GFAP (diluted 1:8) for 10 min at 37°C followed by biotinylated goat anti-rabbit Ig (mouse/rat adsorbed) for 8 min and then counterstained with hematoxylin for 4 min. Sections were visualized and digitally photographed using an Olympus BX60 microscope equipped with a cooled charge-coupled diode camera.

### Glycoform ratios.

Western blots of 10% brain homogenates of infected mice (*n* = 5 per group) were analyzed by densiometric analysis (Quantity One). Di-, mono-, and unglycosylated banding intensities were calculated as percentages of the total density of each PK-treated sample. The averages of the results from three replicate samples were plotted on a triplot based on calculated glycoform ratios.

### Conformational stability assay.

Conformational stability assays were modified from a previous protocol (15). Aliquots of brain homogenates (15 μl) from each mouse of each group (*n* = 5 per group) were incubated for 1 h at room temperature with increasing concentrations of guanidine hydrochloride (GdnHCl; Sigma) ranging from 0 to 5 M in 0.5 M increments. Samples were precipitated in ice-cold methanol at −20°C overnight and centrifuged at 13,000 × *g* for 30 min at 4°C. Pellets were washed in PMCA buffer and centrifuged three times, resuspended in 18 μl of PMCA buffer, subjected to PK digestion, and Western blotted. Conformational stability was quantified by densitometric analyses of Western blots of triplicate samples from each mouse of each group, plotting the mean percentage of PrP^RES^ remaining ± standard deviation (SD) as a function of GdnHCl concentration and using fourth-order polynomial equations and nonlinear regression (GraphPad Prism) to fit denaturation curves for each prion strain.

### Lesion profiling.

Brain lesion profiling was performed as previously described (15) with slight modifications. Ten neuroanatomic regions were identified in coronal brain sections from all 5 mice of each group as follows: region 1, dorsal medulla; region 2, cerebellum; region 3, superior colliculus; region 4, reticular formation; region 5, hypothalamus; region 6, hippocampus; region 7, thalamus; region 8, cerebral cortex; region 9, primary somatosensory cortex; region 10, caudate-putamen. Pathologists blind to the group identification scored each region for vacuolation, astrogliosis, and PrP^RES^ deposition using the following severity scale: 0, normal; 1, mild; 2, moderate, 3, significant; 4, severe. The average of the sum of the three scores constitutes the severity score for each region.

### Statistical analyses.

One-way analysis of variance (ANOVA) with Tukey posttest analysis was performed using GraphPad Prism.
